# Evaluation of black grape pomace, a fruit juice by‐product, in shalgam juice production: Effect on phenolic compounds, anthocyanins, resveratrol, tannin, and in vitro antioxidant activity

**DOI:** 10.1002/fsn3.4104

**Published:** 2024-03-25

**Authors:** Mehmet Akbulut, Hacer Çoklar, Ayşe Nur Bulut, Said Reza Hosseini

**Affiliations:** ^1^ Department of Food Engineering, Agriculture Faculty Selcuk University Konya Turkey; ^2^ Department of Food Engineering, Akşehir Faculty of Engineering and Architecture Selcuk University Konya Turkey

**Keywords:** anthocyanins, fermented black carrot juice, lactic acid fermentation, resveratrol, tannin, waste utilization

## Abstract

The aims of this research were to investigate the usability of black grape pomace in the production of shalgam juice, which is a traditional fermented Turkish beverage, to transform the pomace into the high value‐added product and to enrich the shalgam juice with phenolic compounds. Black grape pomace and black carrot were used as the sources of polyphenols and five different formulations were obtained according to the amounts of black carrot and black grape pomace. During the fermentation, the samples were taken at different periods and analyzed for anthocyanins, phenolic compounds, antioxidant activity, and tannin content. Gentisic, caffeic, ferulic, coumaric, and chlorogenic acids, catechin, glucosides of kaemferol and isorhamnetin, resveratrol, rutin, cyanidin‐3‐xylosylglucosylgalactoside, cyanidin‐3‐xylosylgalactoside, cyanidin‐3‐xylosylglucosylgalactoside acylated with sinapic acid, ferulic acid, or coumaric acid, and glucosides of cyanidin, petunidin, and malvidin were identified in the shalgam juices that contained both black grape pomace and black carrot in their formulation. Some of these polyphenols were not detected detect in the shalgam juices that were produced from only the black carrot or black grape pomace. During the fermentation, a decrease in the amount of anthocyanins originated from black carrots and an increase in the amount of anthocyanins orginated from black grape pomace were determined. Black grape pomace addition to the formulation before the fermentation caused an increase in the amount of tannin in the shalgam juice samples. Consequently, it is thought that black grape pomace can be fruitfully evaluated in shalgam juice production and can be enhanced by polyphenolic profile of shalgam juice.

## Graphical abstract

The phenolic diversity of traditional shalgam juice was increased in terms of phenolic compounds, such as delphinidin, cyanidin, petunidin, malvidin, peonidin, kaempferol and isorhamnetin glycosides, and resveratrol and catechin by using black grape pomace. The total phenolic contents and ABTS (2,2′‐azino‐bis(3‐ethylbenzothiazoline‐6‐sulfonic acid)) antioxidant activity increased only in the shalgam juices obtained with the black grape pomace throughout the fermentation, except for the 24th day of fermentation. There was a strong relationship between total phenolic content and ABTS antioxidant activity.

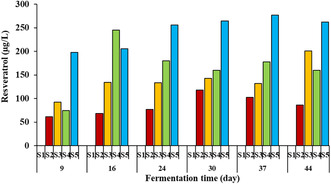

## 1 INTRODUCTION

Grape (*Vitis* spp.) is a widely cultivated and consumed fruit around the world. Worldwide production of grape is almost 80 million tons in 2018, representing 9% of total fruit production (FAOSTAT, 2021). It is consumed as fresh fruit or after processed into juice, wine, molasses, and raisin. During the processing of grapes into juice and wine, skin and seeds are removed as waste. These waste materials of grapes (skin and seeds) are the main constituents of grape pomace. The pomace consists of approximately 40% seeds and 60% grape skins. Grape pomace contains fructose, glucose, protein, soluble and insoluble dietary fiber, vitamin C, fatty acids, polyphenolic compounds, and minerals (Ca, Mg, Na, K, Fe, Mn, P, S, and Zn) (Iora et al., 2015; Sousa et al., 2014).

The increase in environmental awareness has tended food manufacturers to recovery, recycling, and upgrading of waste. Food processing wastes are generally evaluated as animal feed, organic fertilizer, and carbon source in the production of ethanol fuel (Abidin et al., 2014; Vaccarino et al., 1992). However, the utilization of waste, principally rich in phenolic compounds in animal nutrition and soil fertilization, is limited because high consumption levels of these compounds impede the function of gastrointestinal bacteria causing a reduction in the performance of ruminants and also lead to inhibit seed germination (Grbović et al., 2020; Smith et al., 2005). In recent years, food manufacturers and scientists are working on alternative waste management methods, such as ethanol production by fermentation.

Shalgam juice is produced by lactic acid fermentation of bulgur flour, salt, sourdough, turnip, black carrot, and water, and is alternatively known as fermented black carrot juice. Shalgam juice has an intense red color, sourish taste, and cloudy appearance. Its red color arises from anthocyanins that are found in the black carrot used in the formulation. It can be said that besides the contribution of the black carrot to the flavor, its main effect is on the color of the juice. Shalgam juice is included in functional foods due to its microbiological flora and bioactive compounds. Its main bioactive compounds are polyphenols that originate from black carrot (Akbulut & Çoklar, 2020; Kahve et al., 2022; Ulucan et al., 2022).

Phenolic profiles of plants can be used as chemical fingerprints to identify plant species (Akbulut & Akbulut, 2023; Areias et al., 2001). Both black carrot and grape have different phenolic compounds. The predominant polyphenols of black carrot are anthocyanins and phenolic acids (Kamiloglu et al., 2018), while red grapes contain flavonols, flavanols, and stilbenes in addition to anthocyanins and phenolic acids (Lingua et al., 2016b). Chlorogenic, *p*‐coumaric, caffeic, and ferulic acids, hydroxybenzoic acid derivatives, cyanidin‐3‐xylosylglucosylgalactoside, cyanidin‐3‐xylosylgalactoside, cyanidin‐3 xylosyl(sinapolyglucosyl)galactoside, cyanidin‐3‐xylosyl(feruloylglucosyl)galactoside, and cyanidin‐3‐xylosyl(coumaroylglucosyl)galactoside are phenolic compounds in black carrot (Kammerer et al., 2004). Black grape pomace contains resveratrol, catechin, epicatechin, epicatechin gallate, kaempferol, myricetin, quercetin, isorhamnetin, isoquercetin, kaempferol‐3‐glucoside, isorhamnetin‐3‐glucoside, quercetin‐3‐glucoside, laricitrin, syringetin, and glucosides of delphinidin, cyanidin, petunidin, peonidin, and malvidin (Lingua et al., 2016a). *trans*‐Resveratrol is the most notable compound of grape polyphenols due to its inhibitory effect on cancer, diabetes, and cardiovascular diseases (Hung et al., 2000; Jang et al., 1997; Szkudelska & Szkudelski, 2010). Different phenolic compounds have also been reported in both black carrot and grape pomace compared to previously investigated cultivars.

The aims of the research were to transform the grape pomace that serves as a waste material in the food industry into a high value‐added product and also enhance the functional properties of shalgam juice by enriching it with phenolic compounds. For this purpose, black grape pomace was added to the formulation as a carrot substitute before the fermentation and the change in phenolic compounds, antioxidant activity, and tannin content during the fermentation was investigated.

## 2 MATERIALS AND METHODS

### 2.1 Materials

Bulgur flour, salt, water, black carrot, black grape pomace (the pomace of Ekşikara black grape variety), and *Saccharomyces cerevisiae* were used in the production of shalgam juice. Black carrots, bulgur flour, salt, and *S. cerevisiae* were obtained from Gunseven Inc. Ekşikara black grape berries were obtained from Hadim‐Konya and their pomace were produced in the pilot plant of Selcuk University Food Engineering Department.

### 2.2 Methods

#### 2.2.1 Fermentation method

Shalgam juice was produced by the two‐stage fermentation method. For this purpose, bulgur (0.91%) was fermented with yeast (0.2%) before main fermentation for 24 h at 28°C in a jar (10 L). After the first fermentation, salt (1.16%) and black carrot (16.6%) were added to the jar and filled with water up to 10 L. The sample prepared according to this formulation was accepted as the control sample. While the amounts of yeast, bulgur flour, salt, and water remained constant in other formulations, black carrot was replaced with black grape pomace at different rates (0%, 25%, 50%, 75%, and 100%). Samples were coded according to the black carrot and grape pomace ratio as S1 (100% black carrot), S2 (75% black carrot + 25% black grape pomace), S3 (50% black carrot + 50% black grape pomace), S4 (25% black carrot + 75% black grape pomace), and S5 (100% black grape pomace). The lids of the jars were tightly closed and left to the fermentation.

The fermentation was carried out at 20°C. The progress of the fermentation was monitored by measuring the total titratable acidity and terminated when the acidity increase ended. After the filtration, the production of shalgam juice was completed and lasted an average of 44 days.

#### 2.2.2 Determination of the total amount of total phenolic compounds

The total amount of phenolic compounds was determined by the Folin–Ciocalteu colorimetric method. Briefly, 2.5 mL of 0.2 N Folin–Ciocalteu and 2 mL of sodium carbonate (75 g/L) solutions were added to the shalgam juice sample (0.5 mL) diluted with ultrapure water. The absorbance was read against pure water at 765 nm in a spectrophotometer after incubation for 2 h at room temperature (Hitachi, UV 1800, Japan). The results were determined according to the calibration curve prepared with the gallic acid standard (Singleton & Rossi, 1965).

#### 2.2.3 Determination of total monomeric anthocyanin content

Total monomeric anthocyanin contents in the shalgam juice samples were determined using the pH differential method described previously (Lee et al., 2008). One milliliter of shalgam juice was transferred to two different tubes. The first tube was diluted with 4 mL of pH 1.0 buffer (potassium chloride, 0.025 M) and the second tube was diluted with pH 4.5 buffer (sodium acetate, 0.4 M), separately. After 30 min, the wavelengths at 515 and 700 nm were measured, and the difference in absorbance was calculated according to Equation 1. The total monomeric anthocyanin contents of the samples were calculated according to Equation 2. Results are expressed in milligrams (mg) of cyanidin‐3‐glucoside/L.

(1) $$A={\left(A_{520\;\mathrm{nm}}-A_{700\;\mathrm{nm}}\right)}_{\mathrm{pH}\;1.0}-{\left(A_{520\;\mathrm{nm}}-A_{700\;\mathrm{nm}}\right)}_{\mathrm{pH}\;4.5}$$

(2) $$\text{Monomeric anthocyanin content}\;\left(\mathrm{mg}/L\right)=\frac{\;\left(A\times \mathrm{MW}\times \mathrm{DF}\times 1000\right)}{\left(\varepsilon \times l\right)}$$

where MW (molecular weight) = 449.2 g/mol for cyanidin‐3‐glucoside; *ε* = 26,900 molar extinction coefficient, in L/mol/cm; *l* = Path length in cm; DF = Dilution factor.

#### 2.2.4 Determination of phenolic profile

Shalgam juice samples were purified before phenolic profile analysis. C18 SEP‐Pak cartridges activated with acidified water, ethyl acetate, and methanol were used for purification. The cartridge was filled with 1 mL of shalgam juice. Non‐phenolic, phenolic compounds, and anthocyanins were eluted by acidified water, ethyl acetate, and methanol, respectively. Ethyl acetate and methanol were removed under vacuum using an evaporator, phenolics and anthocyanins were dissolved in methanol and transferred to a vial after filtering using a 0.45‐μm syringe (Coklar & Akbulut, 2017).

Phenolic compounds were detected by high‐performance liquid chromatography with diode array detection (HPLC‐DAD) (Agilent 1260 Infinity Series, Waldbronn, Germany). Separation was performed on a C18 column (5 μm, 250 × 4.6 mm i.d.) and the flow rate was 0.75 mL/min, and the gradient was as follows: 10%–14% B (5 min), 14%–23% B (11 min), 23%–35% B (5 min), 35%–40% B (14 min), 40%–100% B (3 min), 100% B isocratic (3 min), 100%–10% B (3 min), and 10% B isocratic (4 min). The detector was set to 280, 320, and 360 nm for non‐anthocyanin phenolics and 520 nm for anthocyanins. Phenolic identification was confirmed by comparing the retention times and the ultraviolet (UV) spectra. The data were analyzed using ChemStation software. *R* ^2^ values of calibration curves of gentisic acid, chlorogenic acid, caffeic acid, ferulic acid, p‐coumaric acid, (+)‐catechin, rutin, kaempferol‐3‐O‐glucoside, isorhamnetin‐3‐O‐glucoside, delphinidin‐3‐O‐glucoside, cyanidin‐3‐O‐glucoside, petunidin‐3‐O‐glucoside, peonidin‐3‐O‐glucoside, and malvidin‐3‐O‐glucoside plotted by different concentrations of these phenolic compounds were found to be 1.000, 0.99909, 0.99997, 0.99997, 1.0000, 0.99999, 0.99969, 0.99984, 0.99996, 0.99988, 0.99989, 0.99989, 0.99997, and 0.99998, respectively.

#### 2.2.5 Resveratrol analysis

Extraction of resveratrol from shalgam juice was carried out by the liquid–liquid extraction method. For this purpose, 120 mL of acetone was added to 40 mL of shalgam juice and shaken well. For phase separation, the mixture was kept at +4°C and the acetone phase was taken. After evaporating the acetone under vacuum, the residue was dissolved in an ethanol:water mixture and transferred to the vial. Resveratrol analysis was performed by HPLC (Agilent, 1260 Series). Separation process was carried out in a C18 column (5 μm, 250 × 4.6 mm). Acetic acid and acetonitrile at a flow rate of 1 mL/min were used as the mobile phase. Detection was performed with a diode array detector (DAD) at 306 nm (Lamuela‐Raventos et al., 1995). *R* ^2^ value of calibration curve of resveratrol by different concentrations of the phenolic compound was 1.000.

#### 2.2.6 Determination of tannin content

Tannin content was determined according to the method based on the principle that standard protein precipitates condensed tannins. Briefly, 2 mL of bovine serum albumin solution (1 mg/mL) was added to 1 mL of shalgam juice, vortexed, and kept at room temperature for 15 min. The mixture was centrifuged at 3000 × *g* for 15 min and the supernatant was removed. The residue accumulated at the bottom of the tube was dissolved in 4 mL of SDS/TEA (5% triethanolamine, 1% sodium dodecyl sulfate) solution, and 1 mL of ferric chloride (FeCl_3_) (0.01 M) was added and instantly vortexed. The absorbance value at 510 nm was read in a spectrophotometer (UV‐1800, Hitachi, Japan). The tannin contents of the samples are given as milligrams (mg) of tannic acid equivalent/L (Hagerman & Butler, 1978).

#### 2.2.7 Determination of antioxidant activity

The DPPH (2,2‐diphenyl‐1‐picrylhydrazyl) antioxidant activity of the samples was determined by measuring the absorbance difference at 515 nm wavelength as a result of radical reduction by antioxidant effective compounds of shalgam juice. For this purpose, 0.1 mL of shalgam juice diluted with pure water was added to 3.9 mL of DPPH solution (6 × 10^−5^ M in methanol). After 30 min of incubation in the dark, the absorbance value was read at 515 nm. The results are given as mmol Trolox equivalents/L (Brand‐Williams et al., 1995).

The ABTS antioxidant activity of samples was determined using the method described by Re et al. (1999). Potassium persulfate solution (2.5 mL, 2.45 mM) was mixed with 5 mL of fresh ABTS stock solution (7 mM) and kept in the dark at ambient temperature for 16 h to generate ABTS^•^. ABTS^•^ stock solution was diluted with ethanol so that the absorbance at 734 nm was 0.700 ± 0.02. ABTS^•^ solution (990 μL) was added to the samples (10 μL) and the absorbance at 734 nm was determined after 6 min of incubation. The results are given as mmol Trolox equivalents/L.

#### 2.2.8 Statistical analysis

The results were given as mean ± standard deviation, and the analysis of variance (ANOVA) was performed to determine whether the effect of grape pomace ratio and fermentation time influenced antioxidant activity, total phenolic substance, tannin content, and anthocyanins and non‐anthocyanin–phenolic compounds. A comparison of the difference between the means was determined by Duncan's multiple comparison test. Minitab (Release 14, Minitab Inc., USA) and Mstat C (Mstat C, 1988) programs were used for statistical analysis.

## 3 RESULTS AND DISCUSSION

### 3.1 Individual phenolic compounds

In this study, 5 phenolic acids, 1 flavan‐3‐ol, 3 flavonol glycosides, 1 stilbene phytoalexin, and 10 anthocyanins were identified in the shalgam juices. Six non‐anthocyanin polyphenolics were detected in the sample formulated with only black carrot (S1), and six different phenolic compounds in the sample formulated with only grape pomace (S5). Due to the chlorogenic acid and rutin found in both black carrot and black grape pomace, 10 non‐anthocyanin phenolic compounds were detected in the shalgam juice that contained both black carrot and black grape pomace.

Gentisic, caffeic, ferulic, and *p*‐coumaric acids were not found in the sample that had only grape pomace in its formulation (S5) (Table 1). The concentrations of these phenolics also increased with increase of the black carrot ratio and the highest value was recorded in the formulation containing 100% carrot (S1).

**TABLE 1:** Phenolic acids (mg/L) detected in fermented black carrot juice.

FD	Samples	Gentisic	Chlorogenic	Caffeic	Ferulic	*p*‐coumaric
9	S1^†^	4.38 ± 1.32^abc^	145.29 ± 7.48^b^	4.67 ± 0.89^b^	6.26 ± 1.56^b^	1.32 ± 0.21^a^
	S2	2.04 ± 0.13^fgh^	143.74 ± 5.88^b^	4.84 ± 0.01^b^	6.70 ± 0.39^b^	0.36 ± 0.20^def^
	S3	2.76 ± 0.73^defg^	71.37 ± 23.84^cde^	2.81 ± 0.24^de^	3.25 ± 0.63^cde^	0.23 ± 0.08^efgh^
	S4	2.40 ± 0.79^efg^	42.67 ± 3.39^efg^	1.69 ± 0.03^fg^	1.77 ± 0.12^ef^	0.12 ± 0.02^fgh^
	S5	nd	1.81 ± 0.42^h^	nd	nd	nd
24	S1	4.54 ± 0.39^abc^	154.58 ± 18.39^b^	5.22 ± 0.46^b^	7.11 ± 0.72^b^	0.81 ± 0.12^b^
	S2	3.47 ± 0.68^cde^	132.20 ± 7.20^b^	4.11 ± 0.18^bc^	6.25 ± 0.21^b^	0.50 ± 0.02^cd^
	S3	2.12 ± 0.60^fgh^	89.86 ± 27.02^c^	3.55 ± 0.22^cd^	4.61 ± 0.45^c^	0.26 ± 0.11^defg^
	S4	1.0 ± 0.07^hı^	31.94 ± 4.72^fg^	1.49 ± 0.12^fg^	2.34 ± 0.47^def^	0.16 ± 0.07^fgh^
	S5	nd	2.11 ± 0.47^h^	nd	nd	nd
37	S1	4.95 ± 0.65^ab^	199.07 ± 11.84^a^	8.00 ± 1.53^a^	12.57 ± 1.85^a^	0.75 ± 0.23^b^
	S2	4.19 ± 0.19^abc^	128.72 ± 8.70^b^	4.18 ± 0.44^bc^	6.37 ± 1.12^b^	0.47 ± 0.03^cde^
	S3	3.74 ± 0.03^bcd^	60.32 ± 26.74^def^	2.39 ± 0.41^ef^	3.18 ± 0.87^cde^	0.12 ± 0.06^fgh^
	S4	2.94 ± 0.55^def^	30.33 ± 4.18^gh^	1.26 ± 0.14^g^	1.59 ± 0.41^efg^	0.09 ± 0.05^gh^
	S5	nd	2.96 ± 2.04^h^	nd	nd	nd
44	S1	5.33 ± 0.40^a^	96.56 ± 8.66^c^	4.73 ± 0.79^b^	6.77 ± 0.28^b^	0.71 ± 0.15^bc^
	S2	4.48 ± 0.39^abc^	83.03 ± 15.59^cd^	3.49 ± 0.09^cd^	3.68 ± 0.75^cd^	0.28 ± 0.01^defg^
	S3	3.76 ± 0.10^bcd^	54.89 ± 11.36^defg^	2.40 ± 0.37^ef^	1.90 ± 0.27^ef^	0.13 ± 0.03^fgh^
	S4	1.58 ± 0.02^gh^	27.43 ± 4.62^gh^	1.88 ± 0.07^efg^	1.36 ± 0.07^fg^	0.05 ± 0.07^gh^
	S5	nd	1.35 ± 1.90^h^	nd	nd	nd

*Note*: Different letters in the same column indicate statistically significant differences between the samples.

Abbreviations: FD, Fermentation periods (day); nd, non‐detectable.

^†^ S1: 100% black carrot, S2: 75% black carrot + 25% grape pomace, S3: 50% black carrot + 50% grape pomace, S4: 25% black carrot + 75% grape pomace, S5: 100% grape pomace.

The order of the phenolic acids according to their concentrations in shalgam juice was noticed as chlorogenic acid > ferulic acid > caffeic acid > gentisic acid > p‐coumaric acid for all samples until the 37th day of the fermentation. After the 37th day, gentisic acid took the place of the caffeic acid and the order of the phenolic acids changed as chlorogenic acid > ferulic acid > gentisic acid > caffeic acid > *p*‐coumaric acid. It was seen that the gensitic, chlorogenic, caffeic, *p*‐coumaric, and ferulic acid contents of shalgam juices were affected by the black grape pomace and black carrot ratio (*p* < .01) and the fermentation time (*p* < .01).

Both individual concentrations and total amounts of phenolic acid were highest in shalgam juice formulated with 100% black carrot, and decreases in their concentrations occurred in proportion to the decrease in black carrot ratio.

Pertaining to the fermentation time, the concentration of gentisic acid increased from the beginning to the end of the fermentation and the highest level of the compound was detected in shalgam juice containing 100% black carrot on the 44th day of fermentation. Unlike the gentisic acid, the highest amounts of chlorogenic, ferulic, caffeic, and *p*‐coumaric acids were determined on the 37th day of fermentation. Decreases in the concentrations of these phenolic acids occurred in the last stage.

The only flavan‐3‐ol detected in the shalgam juices was catechin. However, the compound was not detected in the samples that were produced from only black carrot (S1). As the black grape pomace ratio increased in the formulation, catechin concentration also increased in the shalgam juice samples (Table 2). As seen in Table 2, the highest catechin level (77.78 ± 5.37 mg/L) was observed in S5 (juice containing only grape pomace) on the 37th day of fermentation. It was also observed that concentration of the compound increased with progressing fermentation. While the highest levels of the compound for all formulations were found on the 37th day of fermentation, decreases occurred after this stage.

**TABLE 2:** Flavan‐3‐ol and flavonol glycosides (mg/L) of fermented black carrot juice.

FD	Samples	Catechin	Rutin	Kaempferol‐3‐O‐glucoside	Isorhamnetin‐3‐O‐glucoside
9	S1^†^	nd	0.51 ± 0.44^h^	nd	nd
	S2	1.43 ± 0.40^ı^	6.34 ± 1.03^e^	0.78 ± 0.04^e^	1.13 ± 0.21
	S3	1.00 ± 0.88^gh^	10.59 ± 1.57^d^	3.83 ± 0.07^ab^	2.08 ± 0.15
	S4	24.59 ± 0.41^e^	16.63 ± 1.56^c^	2.95 ± 0.37^cd^	2.79 ± 0.11
	S5	24.52 ± 0.53^e^	22.08 ± 0.06^b^	4.11 ± 0.47^a^	3.68 ± 0.23
24	S1	nd	0.20 ± 0.01^h^	nd	nd
	S2	4.98 ± 1.41^hı^	4.71 ± 2.28^ef^	0.53 ± 0.04^e^	1.18 ± 0.14
	S3	11.63 ± 1.32^gh^	3.70 ± 1.08^efg^	2.83 ± 1.34^cd^	1.80 ± 0.22
	S4	32.88 ± 4.68^d^	16.12 ± 1.52^c^	2.95 ± 0.37^bcd^	2.83 ± 0.38
	S5	51.42 ± 7.62^bc^	22.04 ± 3.26^b^	4.27 ± 0.25^a^	3.54 ± 0.12
37	S1	nd	1.31 ± 0.29^gh^	nd	nd
	S2	10.44 ± 3.36^gh^	1.71 ± 0.21^fgh^	0.08 ± 0.01^e^	1.07 ± 0.08
	S3	20.51 ± 1.58^ef^	2.53 ± 0.32^fgh^	0.29 ± 0.06^e^	1.23 ± 0.03
	S4	57.13 ± 0.85^b^	10.03 ± 1.25^d^	0.72 ± 0.12^e^	2.94 ± 0.64
	S5	77.78 ± 5.37^a^	16.77 ± 1.29^c^	3.00 ± 0.41^bc^	3.03 ± 1.33
44	S1	nd	1.06 ± 0.01^gh^	nd	nd
	S2	1.76 ± 0.27^ı^	2.85 ± 1.20^fgh^	0.07 ± 0.03^e^	0.36 ± 0.28
	S3	16.91 ± 3.26^fg^	2.28 ± 0.34^fgh^	0.32 ± 0.10^e^	0.96 ± 0.03
	S4	45.57 ± 3.77^c^	16.70 ± 1.08^c^	0.14 ± 0.01^e^	1.50 ± 0.25
	S5	49.40 ± 6.35^c^	29.50 ± 1.42^a^	2.07 ± 0.61^d^	16.63 ± 19.45

*Note*: Different letters in the same column indicate statistically significant differences between the samples.

Abbreviations: FD, Fermentation periods (day); nd, non‐detectable.

^†^ S1: 100% black carrot, S2: 75% black carrot + 25% grape pomace, S3: 50% black carrot + 50% grape pomace, S4: 25% black carrot + 75% grape pomace, S5: 100% grape pomace.

Among flavanol glycosides, rutin was the main flavanol glycoside in all shalgam juice samples throughout the fermentation. It was detected in all samples and this indicated that both black carrot and black grape pomace contain rutin at different levels.

The lowest rutin content was determined in the juices that were formulated only with the black carrot (S1) in the first 2 weeks of fermentation. It was also found that as the black grape pomace ratio increased in the formulation, rutin content also increased. Similar to the result of catechin, kaempferol‐3‐O‐glucoside and isorhamnetin‐3‐O‐glucoside were found only in the formulations that contained black grape pomaces.

**FIGURE 1:**
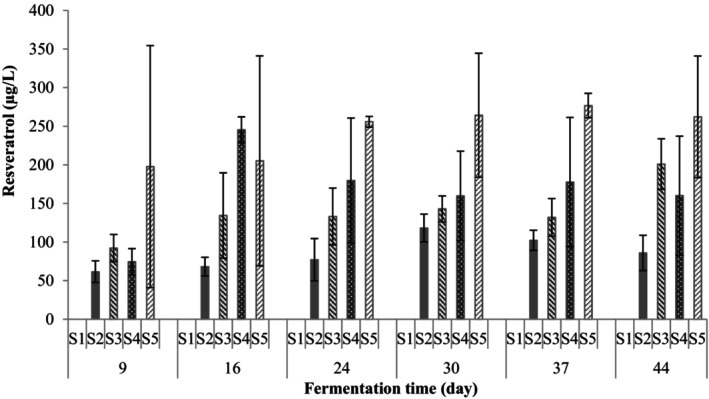
Resveratrol content of fermented black carrot juices (S1: 100% black carrot, S2: 75% black carrot + 25% grape pomace, and S3: 50% black carrot + 50% grape pomace, S4: 25% black carrot + 75% grape pomace, S5: 100% grape pomace).

From Figure 1, the amounts of resveratrol in the shalgam juices during the fermentation are illustrated. According to the results, resveratrol was not detected in the shalgam juice that was formulated only with a black carrot (S1). Furthermore, it was revealed that the amount of resveratrol increased as the grape pomace ratio increased. The resveratrol contents on the 9th day of fermentation were found to be 62, 92, 75, and 198 μg/L for the juices containing 25% black grape pomace (S2), 50% black grape pomace (S3), 75% black grape pomace (S4), and 100% black grape pomace (S5), respectively. In general, increases in the amount of resveratrol for all ratios, except for the 100% black carrot, were observed toward the end of fermentation.

Phenolic biosynthesis in a plant is genetically controlled by enzymes. Therefore, the phenolic profile of a plant can be thought of as its fingerprint. For this reason, it is acceptable that both black carrot and grape contain different polyphenols. The main non‐anthocyanin phenolic compound of black carrot is chlorogenic acid and its concentration varies between 305.6 and 18,790 mg/kg depending on the cultivars (Kammerer et al., 2004; Sun et al., 2009). The black grape pomace contains lower amounts of chlorogenic acid than black carrot. Previous studies have reported the chlorogenic acid concentration in the grape pomace as 38.5–97.75 mg/kg (Coklar, 2017; Doshi et al., 2015).

In previous studies, along with the chlorogenic acid, *p*‐coumaric, caffeic, and ferulic acids and their esters were also reported in black carrot (Kammerer et al., 2004). Kammerer et al. (2004) have reported caffeic acid concentration as 55.2 ± 1.9 mg/100 g dry weight in black carrot.

Although phenolic acids are the main phenolic compounds of black carrot in terms of the concentration, flavanoids are also present in lower amounts. Previous studies have reported quercetin, luteolin, kaemferol, myricetin, and their glucosides in black carrot (Akhtar et al., 2017). Conversely, grape contains higher amounts of flavanoids, particularly flavan‐3‐ol, than phenolic acids. Glycosides of quercetin and kaemferol, monomers and dimers of (+)‐catechin, (−)‐epicatechin, rutin, resveratrol, and chlorojenic acids are some polyphenols detected in grape pomace (Coklar, 2017; Rockenbach et al., 2011). Grape is a significant source of resveratrol and the compound is especially found in the skin of the fruit. Individual anthocyanins of shalgam juice samples and their amounts are shown in Tables 3 and 4. The amounts of anthocyanins were given as cyanidin‐3‐glucoside equivalent due to the lack of standards of some anthocyanins. Cyanidin‐3‐xylosyl(feruloylglucosyl)galactoside, cyanidin‐3‐xylosylgalactoside, cyanidin‐3‐xylosyl(coumaroylglucosyl)galactoside, cyanidin‐3‐xylosylglucosylgalactoside, and cyanidin‐3‐xylosyl(sinapolyglucosyl)galactoside were detected in descending order in the sample containing 100% black carrot (S1). In addition to these anthocyanins, the glucosides of cyanidin, petunidin, and malvidin were detected in the black grape pomace‐added samples. During the fermentation, the amounts of anthocyanins originated from the black carrot increased until the 37th day of fermentation and decreased after this period. Delphinidin‐3‐O‐glucoside and peonidin‐3‐O‐glucoside were detected only in the S5 sample. Similar to carrot originated anthocyanins, the amounts of grape pomace originated anthocyanins decreased after the 37th day of fermentation.

**TABLE 3:** Anthocyanins (mg/L) of fermented black carrot juice.

FD	Samples	1	2	3	4	5
9	S1^†^	31.15 ± 1.55^b^	119.64 ± 7.81	23.47 ± 3.93	168.09 ± 19.61	55.56 ± 1.00^b^
	S2	20.89 ± 1.39^d^	68.13 ± 10.67	13.81 ± 6.34	109.21 ± 6.49	38.45 ± 4.40^cd^
	S3	18.60 ± 0.68^de^	45.18 ± 3.82	9.09 ± 1.38	60.86 ± 4.09	32.92 ± 2.00^cde^
	S4	16.32 ± 4.62^def^	24.90 ± 2.10	4.71 ± 1.54	30.72 ± 0.85	25.23 ± 0.48^efg^
	S5	nd	nd	nd	nd	nd
24	S1	39.31 ± 1.29^a^	130.10 ± 0.68	28.46 ± 1.96	173.40 ± 6.45	58.03 ± 2.45^b^
	S2	28.86 ± 3.36^bc^	81.24 ± 5.05	20.39 ± 1.51	114.61 ± 6.75	40.25 ± 1.92^c^
	S3	16.53 ± 1.28^def^	44.63 ± 3.84	10.47 ± 1.80	62.58 ± 2.11	30.28 ± 1.81^cdef^
	S4	13.60 ± 1.96^efg^	22.94 ± 1.04	4.29 ± 2.09	27.80 ± 2.18	15.47 ± 3.11^ghı^
	S5	nd	nd	nd	nd	nd
37	S1	39.41 ± 6.07^a^	141.87 ± 25.08	32.02 ± 6.07	202.56 ± 28.90	69.55 ± 17.73^a^
	S2	25.93 ± 0.03^c^	75.62 ± 2.30	20.97 ± 1.70	119.62 ± 10.51	39.86 ± 3.23^c^
	S3	11.50 ± 1.41^fg^	31.47 ± 0.76	7.69 ± 0.28	48.99 ± 3.35	21.70 ± 3.72^fgh^
	S4	9.67 ± 2.00^gh^	20.61 ± 0.56	4.19 ± 2.05	28.00 ± 0.53	11.43 ± 1.13^hı^
	S5	nd	nd	nd	nd	nd
44	S1	31.47 ± 2.06^b^	114.54 ± 4.49	25.57 ± 0.12	164.28 ± 13.14	53.00 ± 0.49^b^
	S2	19.36 ± 0.40^d^	62.21 ± 0.67	14.94 ± 0.90	91.72 ± 3.01	28.96 ± 0.07^def^
	S3	9.17 ± 1.75^gh^	31.50 ± 4.80	5.99 ± 1.38	45.64 ± 5.67	19.72 ± 5.06^fghı^
	S4	6.19 ± 1.22^h^	17.96 ± 0.01	3.16 ± 1.69	24.89 ± 0.81	9.22 ± 0.4^ıj^
	S5	nd	nd	nd	nd	nd

*Note*: 1: Cyanidin‐3‐xylosylglucosylgalactoside; 2: cyanidin‐3‐xylosylgalactoside; 3: cyanidin‐3‐xylosyl(sinapolyglucosyl)galactoside; 4: cyanidin‐3‐xylosyl(feruloylglucosyl)galactoside; and 5: cyanidin‐3‐xylosyl(coumaroylglucosyl)galactoside. Different letters in the same column indicate statistically significant differences between the samples.

Abbreviations: FD, Fermentation periods (day); nd, non‐detectable.

^†^ S1: 100% black carrot, S2: 75% black carrot + 25% grape pomace, S3: 50% black carrot + 50% grape pomace, S4: 25% black carrot + 75% grape pomace, S5: 100% grape pomace.

**TABLE 4:** The other anthocyanins (mg/L) of fermented black carrot juice.

FD	Samples	6	7	8	9	10
9	S1^†^	nd	nd	nd	nd	nd
	S2	nd	1.00 ± 0.05^bcde^	2.20 ± 0.43^ef^	nd	20.97 ± 6.22^de^
	S3	nd	0.84 ± 0.28^bcde^	6.14 ± 0.46^cdef^	nd	44.14 ± 4.37^b^
	S4	nd	1.70 ± 0.84^b^	11.09 ± 2.24^bc^	nd	65.72 ± 6.16^a^
	S5	13.73 ± 014^a^	2.75 ± 0.03^a^	16.84 ± 2.36^a^	19.06 ± 2.40^a^	66.46 ± 12.28^a^
24	S1	nd	nd	nd	nd	nd
	S2	nd	1.47 ± 0.19^bc^	2.52 ± 0.34^ef^	nd	22.71 ± 6.04^de^
	S3	nd	0.85 ± 0.17^bcde^	3.17 ± 0.77d^ef^	nd	39.01 ± 5.86^bc^
	S4	nd	0.78 ± 0.35^bcde^	8.23 ± 1.55^bcd^	nd	48.25 ± 1.61^b^
	S5	10.10 ± 2.63^b^	1.37 ± 0.21^bcd^	13.62 ± 3.42^ab^	11.19 ± 1.n6^b^	61.09 ± 17.71^a^
37	S1	nd	nd	nd	nd	nd
	S2	nd	1.52 ± 0.12^bc^	1.71 ± 0.36^ef^	nd	16.17 ± 2.89^de^
	S3	nd	0.43 ± 0.03^de^	1.39 ± 013^ef^	nd	23.89 ± 0.08^de^
	S4	nd	0.23 ± 0.05^e^	4.93 ± 0.87^def^	nd	38.66 ± 0.49^bc^
	S5	4.21 ± 0.22^c^	0.61 ± 0.08^cde^	6.83 ± 1.40^cde^	5.25 ± 1.1^c^	40.90 ± 0.02^bc^
44	S1	nd	nd	nd	nd	nd
	S2	nd	0.80 ± 0.02^bcde^	1.74 ± 0.67^ef^	nd	13.62 ± 7.19^ef^
	S3	nd	0.20 ± 0.16^e^	0.92 ± 0.30^f^	nd	18.71 ± 0.64^de^
	S4	nd	0.03 ± 0.03^e^	2.35 ± 0.58^ef^	nd	28.52 ± 1.17^cd^
	S5	3.78 ± 2.79^c^	0.75 ± 0.00^bcde^	6.77 ± 1.42^cde^	5.91 ± 2.17^c^	37.70 ± 0.52^bc^

*Note*: 6: Delphinidin‐3‐O‐glucoside; 7: cyanidin‐3‐O‐glucoside; 8: petunidin‐3‐O‐glucoside; 9: peonidin‐3‐O‐glucoside; 10: malvidin‐3‐O‐glucoside. Different letters in the same column indicate statistically significant differences between the samples.

Abbreviations: FD, Fermentation periods (day); nd, non‐detectable.

^†^ S1: 100% black carrot, S2: 75% black carrot + 25% grape pomace, S3: 50% black carrot + 50% grape pomace, S4: 25% black carrot + 75% grape pomace, S5: 100% grape pomace.

During the fermentation, the amounts of malvidin‐3‐O‐glucoside originated from black grape pomace decreased. These results are consistent with findings that malvidin‐3‐O‐glucoside is degraded by *Bacillus lactis*, *Lactobacillus plantarum*, and *Lactobacillus casei* during the fermentation (Ávila et al., 2009).

In this study, ingredients, such as black carrot and black grape pomace used in shalgam production, were kept in the fermentation medium throughout the fermentation. Therefore, anthocyanins found in the black carrots and the black grape pomace continued to pass into the shalgam juice throughout the fermentation. It is thought that this could be the reason for the increase in anthocyanins during the fermentation up to 37th day. In addition, the other reason for the increase in phenolic compounds, such as anthocyanins and catechins up to the 37th day, could be that microorganism activity continues, and microbial enzymes convert the phenolics in bound form in plant tissues into water‐soluble free forms. Toktaş et al. (2018) stated that total flavonoid, total phenolic, and anthocyanin contents and total antioxidant capacity in shalgam juices increased throughout the 24‐day fermentation process. Markkinen et al. (2019) stated that flavonol glycosides in sea buckthorn and anthocyanins in chokeberry were not affected by lactic acid fermentation. Ryu et al. (2019) observed that fluctuations occurred in phenolic compounds and anthocyanins during lactic acid fermentation of blueberry extracts, and some phenolics decreased until the 3rd day of 7‐day fermentation but increased on the 7th day.

Previous studies have stated that fermentation has a reducing effect on anthocyanins and this reductive effect notably changes according to the type of fermentation (Hornedo‐Ortega et al., 2017; Li et al., 2022; Zhu et al., 2022). Hornedo‐Ortega et al. (2017) have reported that acetic fermentation produces higher losses than alcoholic fermentation on the anthocyanin composition of strawberry. Individual anthocynins are affected by fermentation to varying degrees due to their chemical structure. Acylated anthocyanins are more stable to fermentation than other anthocynins and minor losses occur during fermentation (Hornedo‐Ortega et al., 2017; Sun et al., 2022).

In this study, some phenolics that were found in the black carrot and/or the black grape pomace were not detected in the shalgam juice samples. In addition to this, the shalgam juices contained lower amounts of each individual phenolic than those in both black carrot and black grape pomace. These findings can be explained by the fact that the water in the formulation causes excessive dilution of phenolic compounds and even some phenols remained below the detection limits.

Fermentation of shalgam juice is a complex process. Both alcoholic and lactic acid fermentation occur due to the activities of yeast and lactic acid bacteria in the media. Microbial interactions and chemical reactions, including the degradation and/or formation of some compounds, occur simultaneously during fermentation. Changes in polyphenolic compounds during the fermentation are also extremely complicated.

Soluble polyphenols can precipitate after binding proteins (Drinkine et al., 2007). Decrease in some phenolics can be explained by the precipitation of the compounds after binding to proteins in the yeast cell wall (Kammerer & Carle, 2009). The polyphenol concentration can also be reduced by microbial enzyme activity and/or polymerization of compounds via aldehyde or private bridges.

Phenolics in the plant move from the cell to the liquid fermentation media by diffusion. Increase in the amounts of some phenolics in the early stages of fermentation probably arises from the diffusion rate of the compounds and/or release of bound phenolics to free forms.

Similar to the results of this study, lower caffeic and *p*‐coumaric acid values were reported in kei‐apple (*Dovyalis caffra* L.) juice (Minnaar et al., 2017) and cherry juice (Filannino et al., 2015) after fermentation by different lactic acid bacteria and *Saccharomyces* species.

*Lactobacillus* (*Lb*.) *plantarum*, *Lb. paracasei* subsp. *paracasei*, *Lb. brevis*, *Lb. fermentum*, *Lb. pentosus*, *Lb. buchneri*, *Lb. delbrueckii*, *Leuconostoc mesenteroides*, and *Pediococcus* spp. are the lactic acid bacteria associated with shalgam juice. *L. plantarum*, *Lb. brevis*, and *Lb. paracasei* subsp. *paracasei* are present in shalgam juice at all fermentation stages (Daskaya‐Dikmen, 2013). *L. plantarum* has an important role in the biotransformation of phenolics in fermented plant‐based food products. While *Lactobacillus reuteri*, *L. fermentum*, and *L. plantarum* hydrolyze the chlorogenic acid to caffeic acid and quinic acid, *L. plantarum*, *L. fermentum*, and *L. reuteri* metabolize caffeic acid to vinyl catechol and dihydrocaffeic acid and *p*‐coumaric acid to *p*‐vinylphenol and phloretic acid (Filannino et al., 2015; Sánchez‐Maldonado et al., 2011). According to Kammerer et al. (2005), phenolics diffuse from grape pomace into the grape juice with different rates during fermentation. Hydroxycinnamic acids diffuse at the first stage of fermentation (before ethanol formation)and then anthocyanins and flavonol glycosides follow them. After ethanol is formed, an increase in flavan‐3‐ol concentration in grape‐must occurs (Nemanic et al., 2002; Zou et al., 2002).

The fluctuations, which occurred in the amounts of some phenolic compounds during the fermentation, could be the impact of both the microbial activities and the variation in the diffusion rates of each polyphenol.

### 3.2 Tannin, total phenolic, total monomeric anthocyanin contents, and antioxidant activity

**FIGURE 2:**
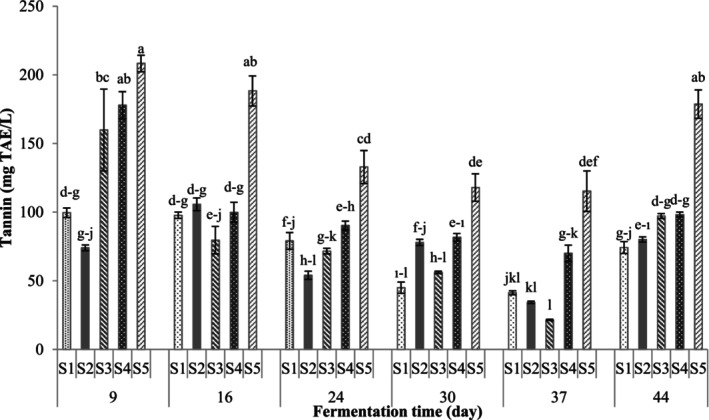
Tannin contents of fermented black carrot juices (S1: 100% black carrot, S2: 75% black carrot + 25% grape pomace, S3: 50% black carrot + 50% grape pomace, S4: 25% black carrot + 75% grape pomace, and S5: 100% grape pomace; Different letters in the same column indicate statistically significant differences between the samples).

Tannin contents of shalgam juices are shown in Figure 2. Increases in the tannin content occurred in the shalgam juices with increasing concentration of the black grape pomace in the formulations. The highest value (208.24 mg TAE/L) was determined at the first stage of fermentation in the sample that contained 100% black grape pomace (S5). The highest levels for all formulations were observed on the 9th day of fermentation. Statistically, significant decreases occurred in the tannin contents of all formulations toward the 37th day of fermentation, while there were increases in the last stage.

Condensed tannins are polymers of proanthocyanins which consist of catechin units and biosynthesized from catechins and anthocyanidins (Mukherjee, 2019). Grapes contain higher amounts of tannins than those of carrots and tannins are found in the seed and skin of grape. Previous studies have reported tannin concentrations in black carrot (Gu et al., 2019) and grape pomace (Carmona‐Jiménez et al., 2018) as 0.22 and 11.02–16.02 mg/g, respectively.

During the fermentation, a strong and negative relationship was seen between the compounds when taking into the consideration tannin and catechin changes in shalgam juices. A decrease in tannin concentration and an increase in catechin concentration occurred simultaneously until the 37th day of fermentation. Conversely, while the amount of tannin increased in all shalgam juice samples at the last stage of fermentation, the amount of catechin decreased. Grape tannins comprise catechins and anthocyanidins. Increment or decrement in the catechin concentration during the fermentation could be as a result of the degradation of tannins, and polymerization of catechin to form tannins. According to the results of Carmona‐Jiménez et al. (2018), *L. plantarum* strains that were isolated from the grape juice show tannase activity. It is crucial to state that *L. plantarum* is one of the most important lactic acid bacteria of shalgam juice flora. Similarly, Shang et al. (2019) have used lactic acid fermetation to remove tannins from Xuan Mugua fruits.

Total phenolic content determined by the Folin–Ciocalteau method and antioxidant activity results are demonstrated in Table 5. According to the data, the total phenolic compounds of shalgam juice containing 100% black carrot (S1) at the end of fermentation were found to be 866.75 ± 28.91 mg GAE/L (gallic acid equivalent per liter). The highest total phenolic compound was found to be 1102.47 ± 45.83 mg GAE/L in the shalgam juice formulated with 100% of black grape pomace (S5) at the last stage. Whereas the lowest value (799.23 ± 18.55 mg GAE/L) was determined in the same sample (S5) on the 24th day of fermentation. At the first two stages of fermentation, higher amounts of total phenolic compounds were determined in the samples with higher amounts of black carrot. Conversely, the shalgam juices containing higher amounts of the black grape pomace had higher phenolic content at the last stages of fermentation. The effects of both black grape pomace ratio (*p* < .01) and fermentation process (*p* < .05) on the total phenolic contents of shalgam juices were statistically significant. Regarding the fermentation duration, it was determined that the total phenolic content increased with ongoing fermentation.

**TABLE 5:** Antioxidant activities, total phenolic compounds, and total monomeric anthocyanin contents of fermented black carrot juices.

FD	Samples	Total phenolic compounds (mg GAE/L)	Total monomeric anthocyanins (mg/L)	DPPH (mmol TE/L)	ABTS (mmol TE/L)
9	S1^†^	927.70 ± 38.74^bcd^	232.43 ± 9.30^a^	3.68 ± 0.15	5.00 ± 0.38
	S2	839.74 ± 70.39^de^	206.96 ± 27.60^ab^	3.32 ± 0.11	4.52 ± 0.09
	S3	864.05 ± 5.45^cde^	201.12 ± 10.77^ab^	3.40 ± 0.21	5.34 ± 0.43
	S4	843.21 ± 18.55^cde^	218.29 ± 4.95^a^	3.03 ± 0.38	6.05 ± 0.22
	S5	876.39 ± 3.27^cde^	200.80 ± 31.59^ab^	3.10 ± 0.20	6.12 ± 1.39
24	S1	867.52 ± 14.73^cde^	220.43 ± 6.49^a^	3.00 ± 0.48	4.78 ± 0.38
	S2	877.55 ± 7.09^cde^	213.33 ± 15.06^a^	3.08 ± 0.28	5.82 ± 0.96
	S3	860.96 ± 27.28^cde^	170.64 ± 16.68^bc^	3.15 ± 0.24	4.73 ± 1.13
	S4	882.95 ± 63.84^cde^	168.10 ± 3.35^bc^	3.37 ± 0.19	5.50 ± 0.69
	S5	799.23 ± 18.55^e^	151.54 ± 23.03^cd^	3.28 ± 0.42	5.86 ± 0.53
37	S1	850.93 ± 18.55^cde^	234.72 ± 35.28^a^	3.08 ± 0.19	5.31 ± 0.90
	S2	879.09 ± 46.37^cde^	195.79 ± 1.48^ab^	3.13 ± 0.29	6.77 ± 0.99
	S3	865.59 ± 13.10^cde^	146.74 ± 2.95^cde^	3.29 ± 0.26	6.51 ± 0.59
	S4	893.36 ± 40.38^bcd^	130.15 ± 14.03^def^	3.33 ± 0.46	6.85 ± 0.50
	S5	970.53 ± 75.30^b^	105.72 ± 3.99^f^	3.25 ± 0.27	7.13 ± 0.93
44	S1	866.75 ± 28.91^cde^	199.03 ± 9.00^ab^	3.08 ± 0.34	4.93 ± 0.04
	S2	877.93 ± 21.82^cde^	172.73 ± 2.21^bc^	2.83 ± 0.12	5.26 ± 0.37
	S3	930.02 ± 1.63^bc^	121.28 ± 8.27^def^	3.07 ± 0.20	6.12 ± 0.60
	S4	867.90 ± 21.82^cde^	111.50 ± 19.54^ef^	3.19 ± 0.19	7.17 ± 0.99
	S5	1.102.47 ± 45.83^a^	100.82 ± 10.63^f^	3.02 ± 0.30	8.63 ± 0.41

*Note*: Different letters in the same column indicate statistically significant differences between the samples.

Abbreviation: FD, Fermentation periods (day).

^†^ S1: 100% black carrot, S2: 75% black carrot + 25% grape pomace, S3: 50% black carrot + 50% grape pomace, S4: 25% black carrot + 75% grape pomace, S5: 100% grape pomace.

Both black carrot and grape pomace are important sources of phenolic compounds. However, the phenolic content of grape pomace is higher than that of black carrot. In earlier studies, total phenolic contents of black carrot (Algarra et al., 2014) and grape pomace (Breksa et al., 2010; Rockenbach et al., 2011) were determined as 1.88–4.92 and 3.16–74.75 mg/g, respectively. In a previous study, the total phenolic contents of berries, seeds, and skin of Ekşikara grape have been reported as 20.91, 115.74, and 49.58 mg GAE/g (Coklar, 2017).

Previous studies have reported increases or decreases in the total phenolic compound concentrations of plants throughout the fermentation (Adetuyi & Ibrahim, 2014; Cuellar‐Álvarez et al., 2017; Weisburger, 2001; Wollgast & Anklam, 2000). In the previous research, it was reported that the fermentation has a decreasing effect on the polyphenolic compounds of cacao beans (Wollgast & Anklam, 2000). Cuellar‐Álvarez et al. (2017) have reported that the total phenolic content of Cupuassu beans increased until the 6th day of fermentation and then decreased toward the end of fermentation. The total phenolic compounds in okra seeds increased with the progress of fermentation (Adetuyi & Ibrahim, 2014).

Losses in phenolic compounds can be attributed to the oxidation of polyphenols by polyphenol oxidase and/or precipitation after forming a water–insoluble protein–polyphenol complex (Cuellar‐Álvarez et al., 2017; Weisburger, 2001). In fermented beverages, the slow diffusion rate of polyphenolic compounds from plant tissues to the liquid fermentation media can prolong the extractability of compounds (Kammerer et al., 2004).

The highest total monomeric anthocyanin amount (234.72 ± 35.28 mg/L) was determined in the S1 sample (containing 100% carrot) on the 37th day of fermentation and the lowest (100.82 ± 10.63 mg/L) in the S5 sample (containing 100% pomace) at the end of fermentation, and it was determined that monomeric anthocyanin amount decreased in all samples with the progress of fermentation. In addition, it was observed that the amount of monomeric anthocyanin decreased with the increase in the black grape pomace ratio in the formulation. The higher values were found in samples containing more black carrot.

Some studies have observed that shalgam juice produced using black carrots increases throughout the fermentation (at the end of 24‐day fermentation). This indicates that the high anthocyanin content in black carrot continues to pass into shalgam juice throughout the fermentation (Toktaş et al., 2018). Different lactic acid bacteria have different effects on anthocyanins. While a decrease was observed in anthocyanins in incubation with *L. plantarum*, *B. lactis*, and *L. casei*, an increase and then a decrease in anthocyanins were observed in incubation with *Lactobacillus acidophilus* (Ávila et al., 2009). The findings we obtained in our study coincide with those of the study of Ávila et al. (2009). This shows that different changes may occur in anthocyanins due to the diversity of microorganism flora in the environment during the lactic acid fermentations.

The highest DPPH value (3.68 ± 0.15 mmol TE/L) was found in the S1 sample on the 9th day of fermentation, and the value decreased to 3.08 ± 0.34 mmol TE/L at the last stage. The shalgam juice that contained 25% black carrot and 75% black grape pomace (S4) possessed the highest DPPH value (3.19 ± 0.19 mmol TE/L) among all samples at the last stage.

When the DPPH and ABTS values in Table 5 were examined, it was determined that the DPPH values of samples decreased throughout the fermentation and the ABTS values increased. Similarly, it was observed that the DPPH value was higher in the samples with a higher black carrot ratio, while the ABTS value was lower. However, the differences between both DPPH and ABTS values were found to be statistically insignificant (*p* > .05).

The ABTS antioxidant activity increased only in the shalgam juices obtained with the black grape pomace throughout the fermentation, except for the 24th day of fermentation. Similarly, in the same samples, an increase in the total phenolic contents was observed throughout the fermentation. In the shalgam juices in which only black carrot was used, both the total phenolic content and ABTS antioxidant activity fluctuated in the direction of increase and decrease throughout the fermentation. This shows that there is a strong relationship between total phenolic content and ABTS antioxidant activity.

Each phenolic compound may possess different antioxidant activity as a result of their chemical structure. Therefore, the profile and concentrations of polyphenols are important in the antioxidant activity of a food product. Heo et al. (2007) have reported that catechin possesses nearly 3 times higher antioxidant activity than chlorogenic acid. On the other hand, Skroza et al. (2015) have reported synergistic or antagonistic interactions between polyphenols. According to their results, there are antagonistic effects between gallic acid–resveratrol, caffeic acid–resveratrol, and quercetin–resveratrol and a synergistic effect between catechin–resveratrol.

## 4 CONCLUSION

Grape pomace was added to shalgam juice formulation and changes in polyphenolic compounds were monitored throughout the fermentation. In addition to gentisic, chlorogenic, caffeic, ferulic, *p*‐coumaric acids, and rutin; resveratrol, catechin, glucosides of kaempferol and isorhamnetin, cyanidin, petunidin, and malvidin were detected in shalgam juices. As grape pomace concentration increased, grape pomace originated phenolic compounds' concentration also increased. While the amounts of total and individual polyphenols changed during the fermentation, there was no statistically significant difference among the antioxidant activities of shalgam juices. In summary, grape pomace, which is considered in the food industry as a waste material, can be added up to 75% in shalgam juice formulations to substitute black carrot in order to enrich the shalgam juice in terms of resveratrol, catechin, rutin, kaempferol‐3‐O‐glucoside, and isorhamnetin‐3‐O‐glucoside, cyanidin‐3‐O‐glucoside, petunidin‐3‐O‐glucoside, and malvidin‐3‐O‐glucoside. The innovative aspect of this study is that the phenolic diversity of traditional shalgam juice was increased in terms of phenolic compounds, such as delphinidin, cyanidin, petunidin, malvidin, peonidin, kaempferol and isorhamnetin glycosides, and resveratrol and catechin by using black grape pomace.

## AUTHOR CONTRIBUTIONS

**Mehmet Akbulut:** Data curation (equal); formal analysis (equal); funding acquisition (equal); investigation (equal); methodology (equal); project administration (equal); software (equal); supervision (equal); writing – review and editing (equal). **Hacer Çoklar:** Formal analysis (equal); investigation (equal); methodology (equal); resources (equal); software (equal); supervision (equal); writing – original draft (equal). **Ayşe Nur Bulut:** Formal analysis (equal); investigation (equal); methodology (equal); software (equal). **Said Reza Hosseini:** Formal analysis (equal); investigation (equal); methodology (equal); software (equal).

## CONFLICT OF INTEREST STATEMENT

The authors declare no conflicts of interest.

## ETHICS STATEMENT

This study does not involve any human or animal testing.

## CONSENT TO PARTICIPATE

Written informed consent was obtained from all study participants.

## Data Availability

The data that support the findings of this study are available on request from the corresponding author.
